# Investigating the Safety of Multiple Body Contouring Procedures in Massive Weight Loss Patients

**DOI:** 10.1007/s00266-022-02941-4

**Published:** 2022-06-01

**Authors:** Saad Mohamed Saad Ibrahiem

**Affiliations:** Department of Plastic Surgery, Reconstructive Surgery, and Burn Management, Faculty of Medicine, Alexandria University, Champollion Street, El-Khartoum Square, Azarita Medical Campus, Alexandria, 21111 Egypt

**Keywords:** MWLP, Combined body countering, Liposuction, Fat transfer, Energy-producing machines, Abdominoplasty

## Abstract

**Introduction:**

Deformities after massive weight loss are usually severe and affect many parts of the body, negatively impacting patients’ social and intimate lives. A common request of patients after massive weight loss is to treat more than one anatomical area in one surgical procedure. Advantages include a single recovery period, lower surgical costs, and faster patient satisfaction. Disadvantages may include increased need for blood transfusions, longer hospital stay, and increased risk of common complications.

**Objective:**

The main objective of the study is to compare operative risk, hospital length of stay, complication rate, and patient satisfaction in MWLP according to the number of surgical procedures performed in the same surgical setting.

**Patients and Methods:**

This is a retrospective case–control study of 653 MWLP who underwent multiple contouring procedures simultaneously in a single surgical procedure. All patients underwent surgery between 2016 and 2020. The patients studied were divided into 4 groups according to the number of anatomical areas operated on.

**Results:**

A total of 1254 body contouring procedures were included in the study. Follow-up time ranged from 13 to 41 months, with a mean of 17 months. The mean age in the study was 33 years old. Women accounted for 78% of the studied population and men accounted for 22%.

The overall complication rate (major and minor) was 105 cases (16.07%) in all groups

**Conclusion:**

Patient satisfaction was highest in patients who underwent 2–3 procedures within the same surgical setting compared to patients who underwent +3 procedures. Nevertheless, this is clinically insignificant.

**Level of Evidence IV:**

This journal requires that authors assign a level of evidence to each submission to which Evidence-Based Medicine rankings are applicable. This excludes Review Articles, Book Reviews, and manuscripts that concern Basic Science, Animal Studies, Cadaver Studies, and Experimental Studies. For a full description of these Evidence-Based Medicine ratings, please refer to the Table of Contents or the online Instructions to Authors www.springer.com/00266.

## Introduction

Obesity is a global epidemic that has various medical problems and also national economic consequences [1]. Bariatric surgery is considered one of the most successful procedures for weight loss and long-term body mass index maintenance [2]. However, the major concern of patients is the excess skin and the resulting deformities after successful massive weight loss. Undoubtedly, this concern negatively affects their social and intimate activity, as the deformities are usually severe and potentially affect any area of the body that represents a plastic surgical challenge [3].

Matarasso et al. [4] noted that the increasing prevalence of successful bariatric surgery has resulted in a growing number of candidates for multiple body contouring procedures and the emergence of a new subspecialty; bariatric plastic surgery. The number of body contouring procedures after massive weight loss has increased by approximately 4000% from 2000 to 2010. This is due to the increasing number of publications explaining the anatomical details and pathological explanation of the deformities, and proposing different algorisms for various surgical contouring techniques after massive weight loss [5].

In addition, the increased number of deformed areas makes the performance of combined (integrated or multistage) procedures in the same surgical setting increasingly important. Al Aly [6] stated that the target of performing such a comprehensive procedure is to bring patients with massive weight loss (MWLP) into a reasonably normal range of body contours.

Treating more than one anatomical area in a surgical setting has positive psychological and physical effects. Patient satisfaction, motivation, and improved ability to maintain optimal body weight increase over time. However, the safety of the combined procedures is controversial [7].

### Objective

The main objective of the study is the compare the operative risk, hospital stay, complication rate, and patient satisfaction in MWLP according to the number of operative procedure(s) performed in the same surgical setting.

### Patients and Methods

This is a retrospective case–control study of patients with massive weight loss (MWLP) who underwent surgery by a single plastic surgeon (S.I.) between January 2016 and December 2020.

The present study was performed according to the Guidelines of Good Clinical Practice and Scientific Committee of the Faculty of Medicine.

The patients studied were divided into 4 groups, according to the number of anatomical areas operated on in the same surgical setting (Table 1).

**Table 1:** The studied groups

Group	Number of the operated anatomical areas	Number of patients
I	One anatomical area	378
II	Two anatomical areas	70
III	Three anatomical areas	106
IV	More than three anatomical areas	99

The inclusion criteria were MWLP (loss of at least 50% of excess weight calculated from ideal body weight), ages > 18 years, BMI < 40 kg/m2, stable body weight over 12 months, pre-operative hemoglobin value > 10 g/dl, and completed follow-up at least 12 months.

Exclusion criteria were the following: patients with BMI above 40 kg/m2, patients with unstable body weight, current smoker patients who refused to quit smoking before the operation, and patients who had not completed follow-up or whose data were incomplete.

A detailed clinical history was obtained, including history of the weight loss, nutritional status, and concomitant medical conditions, with special attention to any thromboembolic predisposition.

Physical examination included measurement of BMI, distribution of fat and skin laxity, skin quality, previous scars, muscle diastasis, and asymmetries. Also, a standard before/after photographic documentation was also obtained.

A surgical plan was developed according to the patients’ main concerns and priorities. A thorough pre-operative anesthesiologic assessment of the patients’ health status was performed, with emphasis on nutritional status and concomitant medical conditions (which should be under control before surgery). Routine pre-operative evaluation included routine blood screening; renal, liver, thyroid function tests, iron, and vitamin B12 levels.

In addition, all patients have consented to blood transfusion and possible re-do in treated areas using the energy-generating machines as an alternative to excision treatment. Pre-operatively, first-generation cephalosporin antibiotics were administrated prophylactically. Warming and protective measures against the risk of DVT included smoking cessation two weeks before and after the operation, low molecular weight heparin injections pre-operatively, and up to 72 hours postoperatively, alternating pneumatic compression of the lower extremities intraoperatively, and compressive stockings postoperatively.

### Position

In the supine position, neck, arm lift, breast surgery, lipoabdominoplasty, thigh liposuction and lift operations were performed individually or in combination. In the prone position, arm, back, thigh liposuction, and buttock augmentation were performed.

### Surgical Technique

The following surgical principles were used in the study;

(I) Performing the maximum possible surgical steps in one session according to the patient’s needs and priorities;
(II) Not sacrificing any tissue by using the possible excess from the redundant areas to correct the deficient areas;
(III) Sparing the scars in unforgiving areas, like the arm, back, and thigh, by the generous use of energy-generating machines;
(IV) Use of excision procedure in areas where the excess skin was massive.
(V) Warming (operating table pads, warming blankets, fluid warmers) must be available during the procedure to maintain a stable core body temperature.
(VI) Care should be taken to protect the extremities with appropriate axillary or shoulder rolls, to protect the eyes, and to protect the neck during positioning.
(VII) Good pain control allows early mobilization to minimize the complications.

Table 2 summarizes the different contouring procedures in the study.

**Table 2:** The distribution of surgical procedures

Procedures	Group I 378 cases	Group II 70 cases	Group III 106 cases	Group IV 99 cases
*Male breast*				
Liposuction	23	6	18	2
Liposuction and circumareolar resection	12	2	5	4
No vertical scar reduction	1	4	1	0
L-shaped excision	3	3	3	3
*Female breast*				
Reduction mammaplasty	59	8	6	9
Augmentation mastopexy (submuscular)	5	4	9	28
*Arm*				
Liposuction	14	26	34	59
Brachioplasty	7	5	6	12
*Abdomen*				
Lipoabdominoplasty	141	31	43	51
Liposuction alone	19	14	33	40
Fleur-de-lis abdominoplasty	2	0	0	0
Belt lipectomy	3	0	2	1
*Back*				
Liposuction	13	22	77	79
Lower back lift	0	0	0	2
Buttock augmentation				
Butt augmentation using fat	32	4	58	79
Butt augmentation using implant	4	0	0	2
*Thigh*				
Liposuction	35	9	19	45
Thigh lift	3	2	4	2
*Leg*				
Calf augmentation using implant	2	0	0	0
Total	378	140	318	418

### Postoperative Care

Patients were transferred to the ward postoperatively. If the operation took more than 4 hours, patients were transferred to HD unit, where they remained for 6 hours after transfer to the ward. In addition, all patients were encouraged to leave in the late afternoon.

Postoperative blood tests were performed on the first day after surgery. No patient was discharged from the hospital with a hemoglobin level less than 10 gm\dl. According to the regulations of the local health authorities, patients could not be discharged from the hospital if the Hb was < 10 gm/dl blood.

Antibiotic therapy was continued until drain removal. Drains were removed if the blood collection was less than 30 cc within 24 h.

## Results

A total of 653 MWL patients who underwent 653 surgeries with 1254 body contouring procedures were enrolled in the study.

Follow-up time ranged from 13 to 41 months and a mean of 17 months. Follow-up was calculated depending on the last communication with the patient.

The study found a significant difference with respect to gender, females were more likely to have body contouring surgery after massive weight loss, women accounted for 78 % of the studied population, and men accounted for 22%.

The average age in the study was 33 years old. Body contouring surgery was common in middle-aged patients in group I (37.8%) and in young in the other three groups: 41.43%, 43.4%, and 41.41% in group II, III, and IV, respectively.

In total, 488 (74.73%) of the patients had a negative history of smoking, however, 165 patients (25.27%) were positive.

Preoperative hemoglobin value varied from 10.14 to 17.5 g/dl, with an average value of 12.85 g/dl.

All contouring procedures were performed in the second year after bariatric surgery (period of weight stabilization).

Before massive weight loss, all patients were morbidly obese with a median BMI of 45.1 kg/m2.

Bariatric surgery was the main method of weight loss in the study:

- 425 (65.08%) sleeve gastrectomy, the most technique used by bariatric surgeons, referring patients to my department.
- 57 (8.72%) gastric bypass.
- 19 (2.91%) gastric band.
- 38 (5.83%) intragastric balloon placement.
- 114 (17.46%) patients had achieved the results through diet and exercise.

The average weight loss in the study was 38.75 kg, ranging from 22 to 90 kg.

The average BMI before body contouring surgery was 29.38, and the average weight before body contouring surgery was 76 kg, ranging from 50 to 116 kg. 63.24% of the formerly morbidly obese patients in the study failed to achieve a “normal” BMI (< 25 kg/m2).

Patients in group IV have a higher pre-operative BMI, and more weight loss, so they are more likely to have problems with excess skin, which makes them interested in treating more anatomical areas in the same surgical setting than the other three groups.

Table 3 presents the demographic data of the study.

**Table 3:** Demographic data of the study

		Group I	Group II	Group III	Group IV
Number of patients		378	70	106	99
Sex	Males	56 (14.81%)	21 (30%)	33 (31.13%)	10 (10.1%)
	Females	322 (85.19%)	49 (70%)	73 (68.87%)	89 (89.9%)
Age (years)	Minimum	19	20	19	20
	Maximum	53	55	57	56
	Median	38	31	31	32
Age groups	18–20	5 (1.3%)	2 (2.86%)	2 (1.89%)	2 (2.02%)
	> 20–30	101 (26.7%)	29 (41.43%)	46 (43.4%)	41 (41.41%)
	> 31–40	121 (32.08%)	24 (34.29%)	36 (33.96%)	33 (33.33%)
	> 41–50	143 (37.8%)	10 (14.29%)	14 (13.21%)	16 (16.16%)
	> 50	8 (2.12%)	5 (7.14%)	8 (7.55%)	6 (6.06%)
Weight (kg)	Minimum	51.2	52	54.8	50
	Maximum	103	110.6	116	109.9
	Median	69	81.7	78.9	75.55
Weight loss (kg)	Minimum	22	16.2	14	44.6
	Maximum	39	40	84	90
	Median	29	35	35.5	56
BMI	Minimum	19	21.5	21.4	20.1
	Maximum	36.5	39.2	38.7	37.3
	Median	29.3	30.5	29	28.7
Preoperative Hb	Minimum	10.6	10.7	10.14	10.8
	Maximum	17.3	16.4	17.1	17.5
	Mean	12.1	13	13.3	13

Surgery was performed on one anatomical site in 378 patients (57.58%), two anatomical sites in 70 patients (10.72%), three anatomical sites in 106 patients (16.23%), and in four or more anatomical sites in 99 patients (15.16%).

The most common operated areas in females were the back, arm, buttock, and abdomen. The most common operated areas in males were the chest, abdomen, and flanks.

The most common surgical procedure was 266 lipoabdominoplasty (21.21%), followed by 191 liposuctions on the back (15.23%), buttock augmentation with autologous fat in 173 patients (13.80%), liposuction on the arms in 133 cases (10.61%), and 82 reduction mammoplasty (6.54%).

Augmentation mastopexy involved 46 (3.67%) breast implants, and buttock augmentation involved 6 buttock implants (0.48%). The amount of liposuction was classified as small (< 500ml) in 154 cases (25.25%), medium (500- < 2000 ml) in 310 cases (50.82%), and large (2000–5000 ml) in 146 cases (23.93%). Energy-generating machines were used in 481 cases (78.85%). The InMode BodyTite device was used in 291 cases and the Renuvion device in 190 cases.

The average operative time in the study was 2.9 hours, with a minimum of 1.2 hours in group I and a maximum of 6.30 in group VI.

The average postoperative hemoglobin concentration was 11.4 g/dL. Twenty-four patients (3.68%) required a blood transfusion during their hospital stay (two units of blood for each patient). The number of blood transfusions was statically significantly higher in the IV group (22 patients) than in the other three groups (*p* value = 0.001). There were no blood transfusions in groups I and II. The study shows that the blood transfusion rate increases by 22 when more than three body contouring procedures are performed in the same surgical setting.

The average hospital stay was 1.25 days; group I patients were discharged the same day of the operation. The other three groups had a minimum hospital stay of one day and a maximum of six days. Hospital stay data were as follows: 1-day stay for 27.6%; 2 days for 44.7%, 72.3% overall; 3 days for 21.8%; and +3-day stay for 5.9% of the total cases.

Table 4 shows the results of clinical data collection.

**Table 4:** Clinical data comparison between the study group and the control group

		Group I 378 cases	Group II 70 cases	Group III 106 cases	Group IV 99 cases
Operative time (H.M)	Median	1 h. 20 min	3	3.15	4.3
	Minimum	50 min	1.25	1.45	3
	Maximum	2 h. 25 min	4.05	5	6.3
Blood transfusion units		0 (0%)	0 (0%)	2 cases (1.9%)	22 cases (22.22%)
Median hospital stay		Discharged on the same day	One day	2 days	2 days
Complication rate		28 (7.41%)	12 (17.14%)	26 (24.53%)	39 (39.39%)
Patient satisfaction rate	Good to excellent	134 (78.9%)	58 (82.86%)	84 (79.25%)	78 (78.78%)
	Average to poor	36 (21.1%)	12 (17.14%)	22 (20.75%)	21 (21.21%)

Patients’ outcome data were collected through clinical examination at follow-up and a WhatsApp interview (patients were sent a questionnaire as a link created using Forms (Office 365 application)). The patients’ responses were sent directly and separately as an excel form to the application, where the statistical analyses were performed.

Statistical analysis was performed using SPSS statistics version 19.0 (IBM Corporation, NY, USA). *P* values of less than 0.05 were considered statistically significant.

The two-way ANOVA test was used to compare the results between the four groups.

Comparison of preoperative and 12-month postoperative Body-Q results showed statistically significant improvement in all four groups.

All patients groups rated discomfort from excess skin significantly higher before surgery.

Average patient satisfaction was rated good to excellent in 522 patients (79.95%) and average to poor in 131 patients (20.05%).

Patient satisfaction was the highest in patients who underwent 2–3 procedures in the same surgical setting, compared to patients who underwent +3 procedures. Nevertheless, this is not clinically significant (*P* value > 0.05).

Table 5 shows the results of the Body-Q questionnaire.

**Table 5:** The Body-Q results

The parameter	The result (0ut 0f 100)			
	Group I	Group II	Group III	Group IV
The body image before surgery	39.3	37.6	37.9	36.7
The body image 12 months after surgery	80.4	82.3	81.8	80.9
Pre-operative information	91.1	92.6	92.1	89.8
Surgeon work and communication	90.3	91.6	91.7	90.8
Medical staff help and behavior	88.8	89.3	90.8	89.8
Office staff information and communication	79.6	81.7	82.6	79.9
Physical activities	84.9	85.7	87.8	89.2
Sexual life	90.4	91.2	90.9	92.4
Psychosocial well-being	89.6	90.9	90.8	91.1

Complications in the study group were divided into major and minor. Major complications included the following: major (> 5 cm) flap necrosis, severe infection, bleeding requiring re-operation, DVT, pulmonary embolism, and death.

Minor complications included seroma, hematoma, small wound breakdown (< 5 cm), burns, and wound infection.

The overall complication rate (major and minor complications) was 105 cases (16.07%) in all groups; the complication rate per group was not significantly different among the first three groups.

The most serious complications in the study occurred in 12 patients (1.84%): three cases of pulmonary embolism (one in the III group and 2 in the IV group) all of whom were admitted to ICU, and later discharged and treated with oral anticoagulant therapy for six months. There were no deaths among the patients in the study. Major wound dehiscence occurred in 8 cases (the wound could not be closed under local anesthesia); all were readmitted to the hospital and closed under general anesthesia after a Penrose drain was placed. One case of significant necrosis of the flap margin after abdominoplasty (necrosis of the flap was greater than 5 cm^2^) was treated by multiple dressings, debridement, and secondary closure.

Minor complications affected 93 patients (14.24%); all were treated conservatively in the out-patient clinic.

Seromas were the most common minor complication in the study; their incidence was 6.74%.

The second most common was minor wound-healing disorders, with an incidence of 3.68%, all of which were successfully treated with conservative measures. There were 14 hematomas (2.14%), all of which occurred in male patients after liposuction for gynecomastia. There were 11 cases (1.68%) of superficial VASER burns that healed conservatively.

Group analysis in the study showed a steady increase in the risk of developing complications according to the number of surgical steps performed in the same surgical setting: 7.41% in group I, 17.14% in group II, 24.53% in group III, and 39.39% in group IV.

Performing 2 to 3 combined cosmetic procedures did not significantly increase the overall complication rates in the study (*P *= 0.73), but four or more combined cosmetic procedures were associated with an increase in the complication rate.

Table 6 shows the complication rate.

**Table 6:** Complication rate

Complication	Group I 378 cases	Group II 70 cases	Group III 106 cases	Group IV 99 cases
Seroma	16	3	13	12
Minor wound-healing problems	6	5	5	8
Major wound dehiscence	2	0	1	5
Flap necrosis > 5 cm ^2^	0	0	1	0
VASER burn	1	2	3	5
Hematoma	3	2	2	7
DVT	0	0	1	2
Total	28 (7.41%)	12 (17.14%)	26 (24.53%)	39 (39.39%)

Secondary procedures were performed in 17 cases (2.6%). These secondary procedures were 10 repetitions of liposuction using the same energy-generating device (liposuction of the arms (6 patients), liposuction on the back (3 cases), and liposuction on the thighs (3 patients)), to achieve a better tightening effect. Three abdominoplasties scars had to be revised because of wide scars and two back lifts in male patients who were dissatisfied with their back liposuction. The revisions help turn poor response into good satisfaction.

**Fig. 1:**
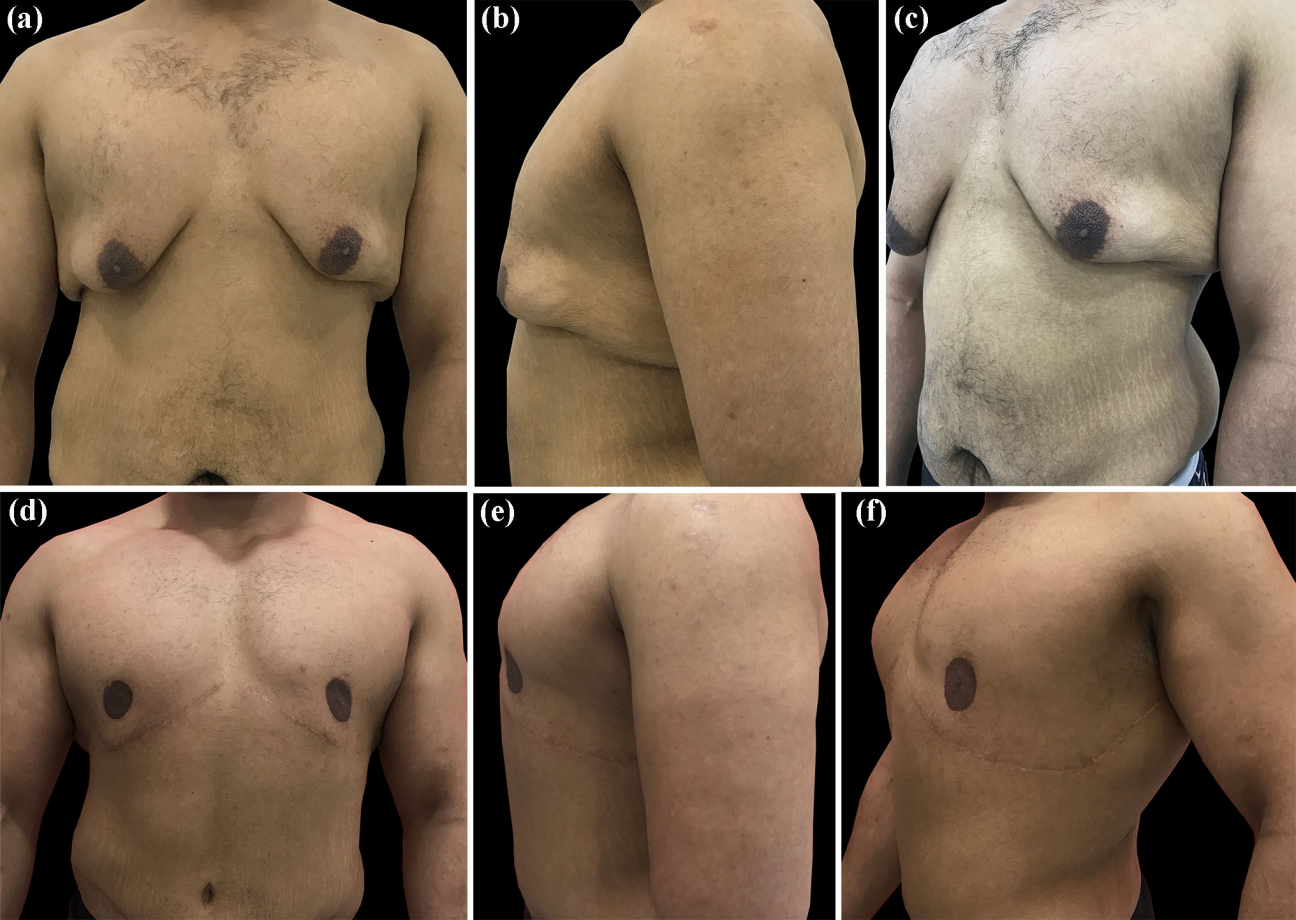
Male patient, 28 years old, 111.7 kg weight, after a weight loss of 63 kg, submitted to a total of two procedures: lipoabdominoplasty and L-shaped gynecomastia excision, with 24-month follow-up. **A**, **B**, **C**: Preoperative anterior, left lateral, and oblique views. **D**, **E**, **F**: Postoperative anterior, left lateral, and oblique views

**Fig. 2:**
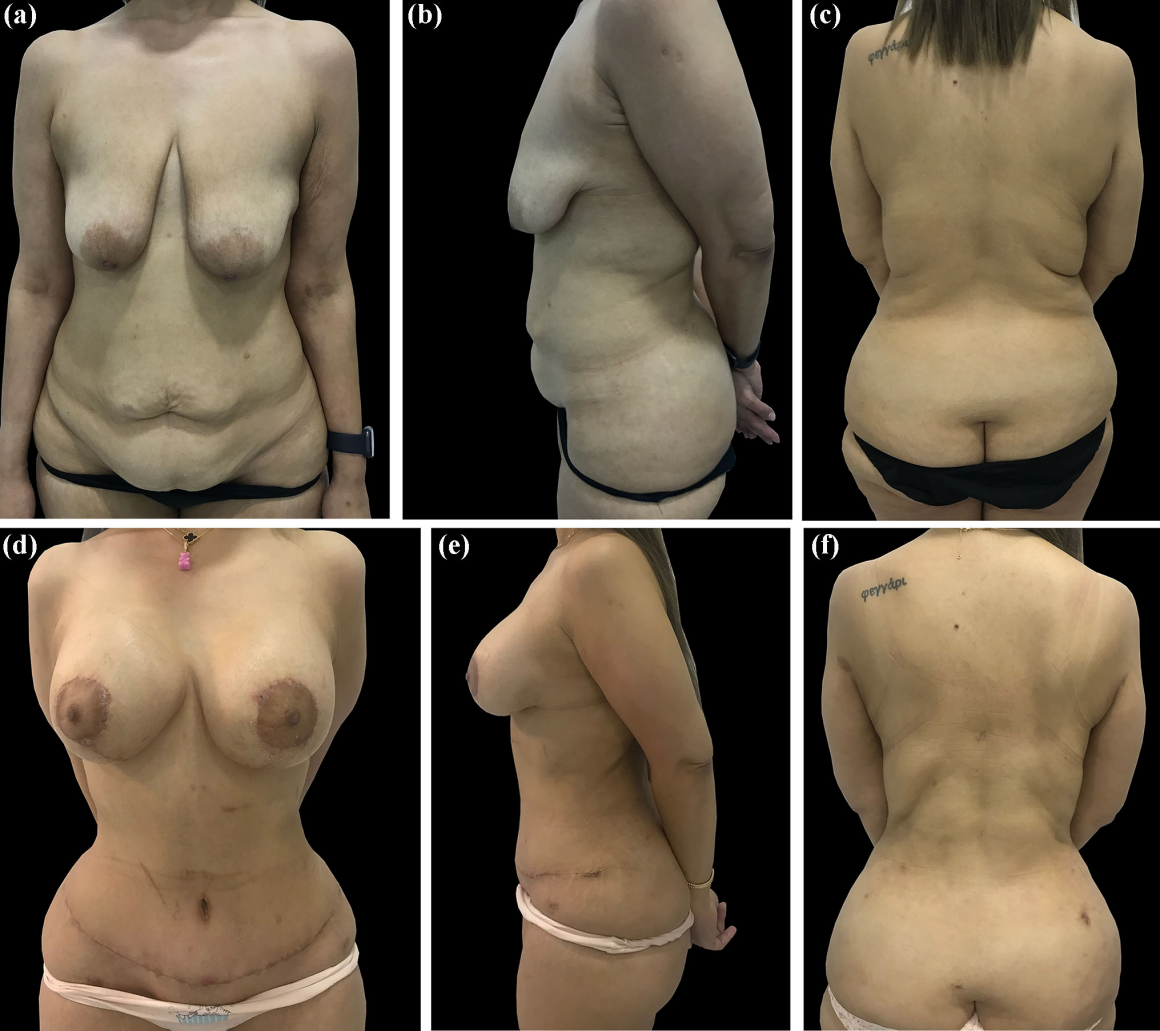
Female patient, 36 years old, 59 kg weight, after a weight loss of 41 kg, submitted to a total of three procedures: lipoabdominoplasty and augmentation mastopexy with micro-textured silicone implant; right side 320cc & left side 320cc; and radio-frequency-assisted liposuction of the back using Bodytite RF platform (Invasix Corp., Yokneam), with 19-month follow-up. **A**, **B**, **C**: preoperative anterior, left lateral, and back views. **D**, **E**, **F**: postoperative anterior, left lateral, and back views

**Fig. 3:**
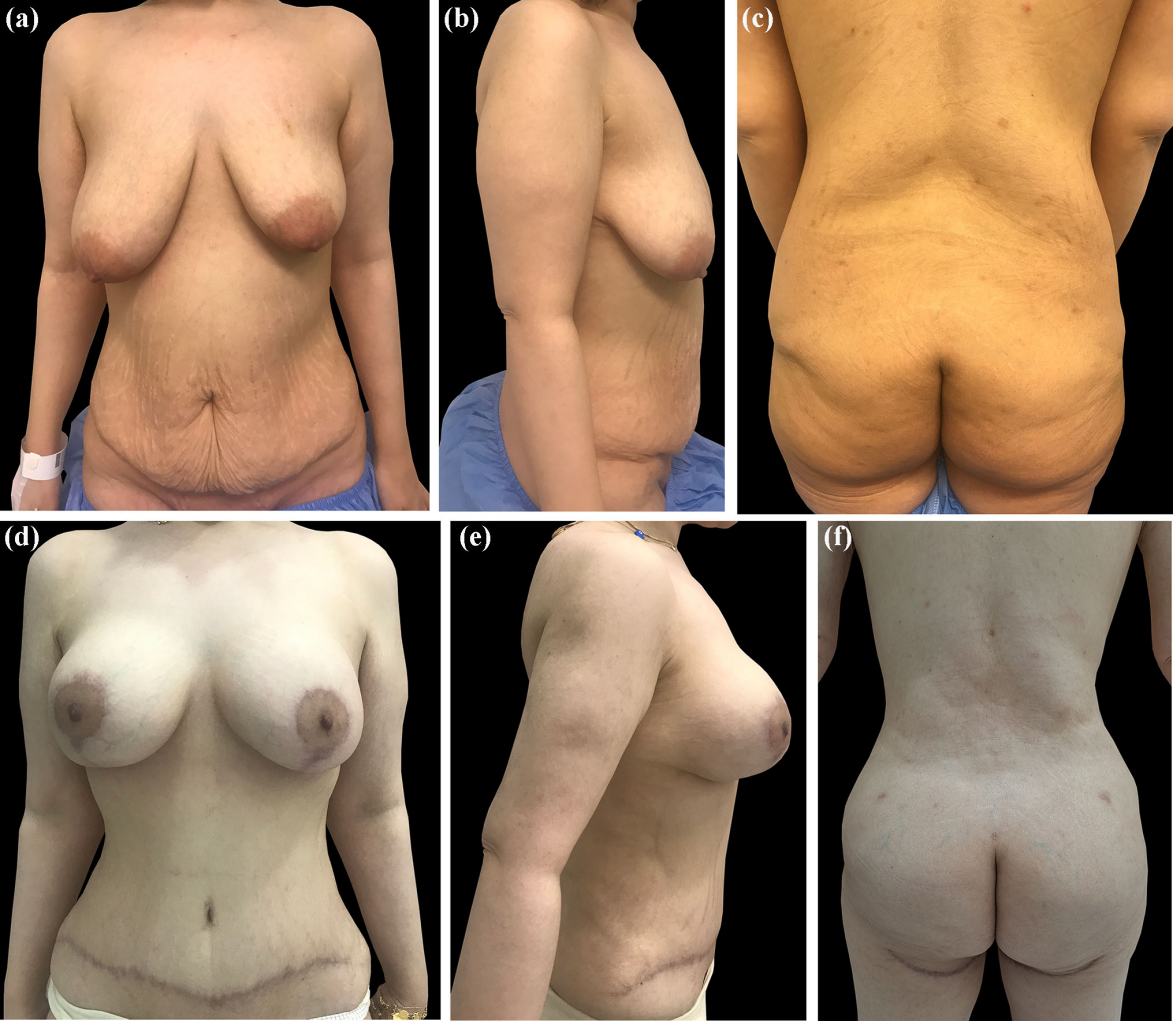
Female patient, 37 years old, 62 kg weight, after a weight loss of 47 kg, submitted to a total of four procedures: lipoabdominoplasty and augmentation mastopexy with micro-textured silicone implant; right side 300 cc & left side 350 cc, radio-frequency-assisted liposuction of the back using Bodytite RF platform (Invasix Corp., Yokneam) and fat transfer to the buttock, with 15-month follow-up. **A**, **B**, **C**: Preoperative anterior, right lateral, and back views. **D**, **E**, **F**: Postoperative anterior, right lateral, and back views

Figures 1, 2 and 3 demonstrate patients at short- and long-term follow-up.

## Discussion

There is no doubt that obesity is a disease of modern times. There are more and more bariatric surgeries targeting obese patients. As a result, the number of patients with multiple deformities in different parts of the body is increasing. Not to mention that the treatment of each body area involves multiple hospitalizations and loss of work and social life. However, the positive effects of body contouring after massive weight loss on body shape, physical functions, and psychological well-being are undeniable [3].

De Castro [8] introduced the term “combined cosmetic surgery” and used it to describe performing of more than one procedure to achieve unrelated aesthetic results during the same operation. So, it is not a new issue, but it is always the question of safety that is a concern. One of the main advantages of the combined approach is the use of excess autologous tissue from one surgical step to augment other areas that require additional tissue, such as breast and buttock areas. This is greatly facilitated when multiple surgical steps are combined in adjacent body areas, provided that good patient selection and preoperative preparation are performed [9].

Many techniques, surgical modifications, and non-surgical energy-generating machines have been introduced for the treatment of various problematic body areas of MWLP. On the other hand, the main concerns of the plastic surgery society were the increased need for blood transfusions and longer hospital stays [10].

Several studies document that MWLP is at a higher risk of complications (hematoma/seroma formation, skin necrosis, infection, and delayed wound healing) after body contouring surgery [11, 12].

Plastic surgeons must be able to predict which patients are at an increased risk of blood transfusion and complications; and how to avoid them by selecting the right patient for the appropriate procedure.

### Blood Transfusion Rate

A blood transfusion should be limited to rare events during combined body contouring in MWL surgery. Many measures can be taken to reduce intraoperative blood loss. These include super wet infusions with epinephrine, careful management of the intraoperative blood pressure (hypotensive anesthesia), adequate medications for pain control, and the use of tranexamic acid. The blood transfusion rate in the study was relatively low (3.68%) compared with similar studies, as 82.54% of the patients studied regularly consumed iron, multivitamins, and trace elements after bariatric surgery. In addition, the average pre-operative hemoglobin level was 12.85 g/dl, which eliminated the need for pre-operative blood transfusions. The average blood transfusion rate was zero in the first three groups. But in IV group, the blood transfusion rate was increased 22-fold.

Modarressi et al. [13] admitted that 8.9% of MWLP required blood transfusion after circular abdominoplasty due to symptomatic anemia (the mean drop of hemoglobin level was 3.8 g/dL.). Hurwitz et al. [10] presented their cases of total body lifts with a mean blood transfusions rate of 1.5 units.

### Hospital Stays

Compared to other series, the average hospital stay in the study was 1.25 days, because all patients in the study paid for their own hospital stay (it was not covered by insurance), and all suction drains were later removed during follow-up visits. De Castro and Daher [8] reported a hospital stay of at least three days in 30% of patients who underwent combined cosmetic surgery. I think the complexity of the procedure is usually not the cause for the long hospital stay, but the complications could be the cause.

### The Complication Rate

Postoperative complications for body contouring surgery in MWLP range from 28% to 78%. However, many complications are minor [14–17].

Several studies have found that pre-bariatric BMI and current BMI are predictors of complications in both multiple contouring procedures after massive weight loss [18].

Marouf and Mortada [19] mentioned a 37% increased risk of complications when pre-body contouring BMI is ≥ 30 kg. Higher resection weight also seems to be associated with a greater risk of complications.

Capella [20] found that patients with higher maximum BMIs before massive weight loss have a higher risk for complications after a body lift. He gives an example that a person with a maximum BMI of 70 kg/m2 before massive weight loss has 15 times higher risk of complications after body lift than someone with a BMI of 40 kg/m2.

Wiser et al. [7] reported a complication rate of 30.6% in MWLP who had surgery on three or more anatomical areas.

In the present study, the overall complication rate was low (16.07%) compared with other studies. Modarressi et al. [13] reported a complication rate of 23.2% after circular abdominoplasty in MWLP, with most complications being minor, in the form of delayed wound healing treated under local anesthesia.

Botero et al [14] have 55.5% complications rate. In total, 13% were major complications and 87% were minor complications.

Pajula et al. [21] identified a total of 96 complications in 80 patients with an overall complication rate of 51%.

There are two common complications of MWLP after performing body contouring surgery: seroma formation and wound-healing problems.

Seroma formation is a common complication due to the extensive tissue undermining in body lift after massive weight loss. It accounts for 5% to 37.5% of all complications [18].

Aly et al. [22] reported an incidence rate of 37.5% of seroma formation. Carwell and Horton [23] described seroma rates of 14%, and Van Geertruyden et al. [24] reported a low seroma rate of 6.6%. In the present study of 653 cases, I had a seroma rate of 6.74%; the minimum time to drain removal was 4 days, and the maximum time was 25 days. I believe that seroma rate could be reduced if the undermining zone in lifting procedures is limited to the resection area. Pascal et al. [25] also believe that using a scalpel instead of electrocautery for incision and dissection could be the reason for their low seroma and wound complication rate.

### Wound-Healing Problems

The second most common complication was minor wound-healing problems, with 3.58% incidence in the present study, while Modarressi et al. [13] stated that 12.5% suffered from localized delayed wound healing.

Several studies mention factors such as malabsorption that can affect healing, as an explanation for the higher rates of wound-healing problems in MWLP [26, 27].

In the presented study, 3 cases of pulmonary embolism were reported as general complications (one case in III group and 2 cases in IV Group), but no deaths were reported.

### Strength of the Study

The strength of this study is the comparability of 4 different groups of MWLP after body contouring surgery, the large sample size (653 patients) with similar demographics, and the comprehensive documentation of complications in all patients.

### Limitation of the Study

Retrospective studies are prone to investigator bias because they depend on medical records review.

## Conclusion

Combined body contouring surgery after massive weight loss is now a standard surgical treatment. It is important to find a balance between the safety of the surgery and the patient’s satisfaction in the medical and psychological fields.

Performing 2 to 3 combined cosmetic procedures in the same surgical setting did not significantly increase the overall complication rates in the study, but four or more combined procedures were associated with an increase in the complication rate.

The high complication in the IV group may be attributed to the amount of excess weight loss, the amount of tissue removed, and the intraoperative hypothermia due to increased operative time.

I believe that many morbidly obese patients do not receive enough information prior to bariatric surgery to prepare them for the physical and psychological changes associated with skin redundancy after successful massive weight loss.

### Recommendation

The author emphasizes the importance of the following:

- Plastic surgeons who routinely perform combined body contouring procedures should have a team. They should have more than one experienced assistant to work harmoniously because harmony in the operating room saves time and effort, which is critical for patient safety; an experienced scrub nurse is essential in body contouring procedures, and a competent circulating staff members is essential for quick and safe positioning of the patient during the procedure, and prompt responses in adjusting the energy-generating machines;
- Careful monitoring of the vital signs and urine output by an experienced anesthesiologist is paramount;
- A long hospital stay does not affect the prognosis of the combined surgery; on the contrary, discharge from the hospital encourages the patient to resume the usual daily activities as soon as possible.

## References

[1] Ogden CL, Carroll MD, Lawman HG (2016). Trends in obesity prevalence among children and adolescents in the United States, 1988–1994 through 2013–2014. JAMA.

[2] Lazzati A, Katsahian S, Maladry D, Gerard E, Gaucher S (2018). Plastic surgery in bariatric patients: a nationwide study of 17,000 patients on the national administrative database. Surg Obes Relat Dis..

[3] Azin A, Zhou C, Jackson T, Cassin S, Sockalingam S, Hawa R (2014). Body contouring surgery after bariatric surgery: a study of cost as a barrier and impact on psychological well-being. Plast Reconstr Surg.

[4] Matarasso A, Aly A, Hurwitz DJ, Lockwood TE (2004). Body contouring after massive weight loss. Aesthet Surg J.

[5] Liu TS, Miller TA (2008). Economic analysis of the future growth of cosmetic surgery procedures. Plast Reconstr Surg.

[6] Aly AS, Cram AE, Heddens C (2004). Truncal body contouring surgery in the massive weight loss patient. Clin Plast Surg.

[7] Wiser I, Heller L, Spector C, Fliss E, Friedman T (2017). Body contouring procedures in three or more anatomical areas are associated with long-term body mass index decrease in massive weight loss patients: a retrospective cohort study. J Plast Reconstr Aesthet Surg.

[8] De Castro C, Daher M (1978). Simultaneous reduction mammaplasty and abdominoplasty. Plast Reconstr Surg.

[9] Hurwitz DJ (2004). Single-staged total body lift after massive weight loss. Ann Plast Surg.

[10] Hurwitz DJ, Ayeni O (2016). Body contouring surgery in the massive weight loss patient. Surg Clin North Am.

[11] Nemerofsky RB, Oliak DA, Capella JF (2006). Body lift: an account of 200 consecutive cases in the massive weight loss patient. Plast Reconstr Surg.

[12] Wilson JA, Clark JJ (2004). Obesity: impediment to postsurgical wound healing. Adv Skin Wound Care.

[13] Modarressi A, Meia Rüegg E, Bezzola T, Pittet-Cuénod B (2016). Circular abdominoplasty after massive weight loss: is it a risky procedure?. J Plast Reconstr Aesthet Surg.

[14] García Botero A, García Wenninger M, Fernández LD (2017). Complications after body contouring surgery in postbariatric patients. Ann Plast Surg.

[15] van der Beek ES, van der Molen AM, van Ramshorst B (2011). Complications after body contouring surgery in post-bariatric patients: the importance of a stable weight close to normal. Obes Facts.

[16] Parvizi D, Friedl H, Wurzer P (2015). A multiple regression analysis of postoperative complications after body-contouring surgery: a retrospective analysis of 205 patients: regression analysis of complications. Obes Surg.

[17] Poodt IG, van Dijk MM, Klein S, Hoogbergen MM (2016). Complications of lower body lift surgery in postbariatric patients. Plast Reconstr Surg Glob Open.

[18] Coon D, Gusenoff JA, Kannan N, El Khoudary SR, Naghshineh N, Rubin JP (2009). Body mass and surgical complications in the postbariatric reconstructive patient: analysis of 511 cases. Ann Surg.

[19] Marouf A, Mortada H (2021). Complications of body contouring surgery in postbariatric patients: a systematic review and meta-analysis. Aesthetic Plast Surg.

[20] Capella JF (2008). Body lift. Clin Plast Surg.

[21] Pajula S, Jyränki J, Tukiainen E, Koljonen V (2019). Complications after lower body contouring surgery due to massive weight loss unaffected by weight loss method. J Plast Reconstr Aesthet Surg.

[22] Aly AS, Cram AE, Chao M, Pang J, McKeon M (2003). Belt lipectomy for circumferential truncal excess: the University of Iowa experience. Plast Reconstr Surg.

[23] Carwell GR, Horton CE (1997). Circumferential torsoplasty. Ann Plast Surg.

[24] Van Geertruyden JP, Vandeweyer E, de Fontaine S, Goldschmidt DP, Duchateau J (1999). Circumferential torsoplasty. Br J Plast Surg.

[25] Pascal JF, Le Louarn C (2002). Remodeling bodylift with high lateral tension. Aesthetic Plast Surg.

[26] Agha-Mohammadi S, Hurwitz DJ (2008). Nutritional deficiency of post-bariatric surgery body contouring patients: what every plastic surgeon should know. Plast Reconstr Surg.

[27] Agha-Mohammadi S, Hurwitz DJ (2010). Enhanced recovery after body-contouring surgery: reducing surgical complication rates by optimizing nutrition. Aesthetic Plast Surg.

